# Role of the fatty pancreatic infiltration in pancreatic oncogenesis

**DOI:** 10.1038/s41598-024-57294-6

**Published:** 2024-03-19

**Authors:** Sonia Frendi, Chloé Martineau, Hélène Cazier, Rémy Nicolle, Anaïs Chassac, Miguel Albuquerque, Jérôme Raffenne, Julie Le Faouder, Valérie Paradis, Jérôme Cros, Anne Couvelard, Vinciane Rebours

**Affiliations:** 1 Inflammation Research Center (CRI), INSERM, U1149, Paris-Cité University, 75018 Paris, France; 2 Pancreatology and digestive oncology Department – DMU Digest, Beaujon Hospital, AP-HP, Paris-Cité University, 100 Boulevard du Général Leclerc, 92110 Clichy, France; 3 INSERM U1149, CNRS ERL 8252, Inflammation Research Center (CRI), Paris-Cité University, 75018 Paris, France; 4 Pathology Department, Bichat Hospital, AP-HP, Paris-Cité University, Paris, France; 5 Pathology Department, FHU MOSAIC, AP-HP, Beaujon Hospital, Clichy, France; 6 JR-Analytics (Jr-Analytics.Fr), 33370 Pompignac, France

**Keywords:** Obesity, Intra-lobular and extra-lobular fatty pancreatic infiltration, Pancreatic precancerous lesions, Lipidomic MALDI-TOF imaging mass spectrometry, Pancreatic oncogenesis, Pancreatic cancer, Cancer, Cell biology

## Abstract

Although pancreatic precancerous lesions are known to be related to obesity and fatty pancreatic infiltration, the mechanisms remain unclear. We assessed the role of fatty infiltration in the process of pancreatic oncogenesis and obesity. A combined transcriptomic, lipidomic and pathological approach was used to explore neoplastic transformations. Intralobular (ILF) and extralobular (ELF) lipidomic profiles were analyzed to search for lipids associated with pancreatic intraepithelial neoplasia (PanINs) and obesity; the effect of ILF and ELF on acinar tissue and the histopathological aspects of pancreatic parenchyma changes in obese (OB) and non-obese patients. This study showed that the lipid composition of ILF was different from that of ELF. ILF was related to obesity and ELF-specific lipids were correlated to PanINs. Acinar cells were shown to have different phenotypes depending on the presence and proximity to ILF in OB patients. Several lipid metabolic pathways, oxidative stress and inflammatory pathways were upregulated in acinar tissue during ILF infiltration in OB patients. Early acinar transformations, called acinar nodules (AN) were linked to obesity but not ELF or ILF suggesting that they are the first reversible precancerous pancreatic lesions to occur in OB patients. On the other hand, the number of PanINs was higher in OB patients and was positively correlated to ILF and ELF scores as well as to fibrosis. Our study suggests that two types of fat infiltration must be distinguished, ELF and ILF. ILF plays a major role in acinar modifications and the development of precancerous lesions associated with obesity, while ELF may play a role in the progression of PDAC.

## Introduction

Pancreatic adenocarcinomas (PDAC) are the fourth cause of cancer mortality in most developed countries^1^. Because of the lack of effective therapies, the 5-year survival rate is still < 9%^2^. The causes of the PDAC pandemic are still not well understood, even if certain constitutional and environmental risk factors have been identified, such as a family history and genetic factors, age, tobacco, chronic pancreatitis, diabetes mellitus, dietary factors and obesity^2,3^.

The prevalence of overweight and obese populations has markedly increased in western countries and has reached pandemic levels, affecting 60–70% of adults. In the past few decades its prevalence has increased by 27% and 47% in the adult and child population, respectively^4^. Obesity is associated with numerous co-morbidities such as diabetes, arterial hypertension and hyperlipidemia (all part of the metabolic syndrome) and an increased risk of cancer, in particular PDAC. In epidemiological studies, the risk ratio of PDAC per 5 kg/m^2^ increase in BMI was 1.07 [0.93–1.23] in men and 1.12 [1.03–1.23] in women^5^.

The physiopathology of obesity and pancreatic cancer is still poorly understood, although some studies have shown the specific roles of subcutaneous, intravisceral fat and pancreatic fat infiltration. In one study using mass spectrometry analyses in patients operated for colorectal cancer, Liesenfeld et al. showed that the distribution of lipids between subcutaneous and visceral fat could be different and that visceral adipose tissue displayed higher levels of inflammatory lipid metabolism markers, such as free arachidonic acid, phospholipases and enzymes involved in prostaglandin synthesis. They also identified lipids in the adipose tissues associated with the tumor^6^.

Obesity and increased intravisceral fat mass induce fatty infiltration in numerous organs including the pancreas. This adipose infiltration includes triglycerides and free fatty acids which play a role in fibrogenesis via sequences of necrosis- as well as direct activation of pro-inflammatory pathways. This pro-inflammatory environment plays a role in the initiation of precancerous pancreatic lesions^7,8^.

The cellular changes that precede the onset of PDAC have not been clearly described. Pancreatic intraepithelial neoplasia (PanIN) is the most commonly described precancerous lesion in PDAC and various studies have shown their acinar origin. A study by Guerra C. et al. based on an inducible KrasG12V mice model showed that acinar or centro-acinar cells could be the origin of PanINs and PDAC by differentiation into ductal-like cells during inflammation^9^. In addition, Kopp JL. et al. showed that PDAC precursor lesions are generated from adult acinar cells by differentiation into ductal-like cells and not from ductal or centro-acinar cells^10^, called acinar-to-ductal metaplasia (ADM). This process is one of the key steps promoting the development of PanINs^11^. Other morphological changes in acinar cells, called acinar nodules (AN), have been described, corresponding to an intermediate state with co-expression of ductal and acinar markers. The relationship of AN to PanINs is unclear^12–14^.

In a previous study our group assessed the prevalence and severity of PanINs according to the type of pancreatic fatty infiltration. We showed that the intra-pancreatic infiltration and fibrosis in OB patients were correlated to BMI and the presence of dysplastic lesions, providing further evidence of the direct link between obesity, chronic pancreatic inflammation and cancer^15^. Two different types of fatty infiltration were observed in the pancreatectomy specimens: intra and extra lobular locations. Both were significantly associated with the presence and/or number of PanINs. We also observed that patients with predominantly intra-visceral fat distribution had more PanIN lesions. On multivariate analysis PanINs were associated with extralobular (ELF) and intralobular (ILF) infiltrations. Other risk factors for PanIN were also found in particular, intralobular fibrosis and a higher BMI^15,16^.

We hypothesize that the composition and pathophysiological role of intra (ILF) and extra lobular fat (ELF) may be different depending on the presence and the stage of precancerous pancreatic lesions (ADM. PanIN) and that pancreatic fatty infiltration plays a key role in the oncogenesis process of the pancreas.

We first evaluated the lipidomic profiles of pancreatic ILF and ELF by MALDI-MSI and searched for specific lipid peaks linked to BMI and PanINs in malignant pancreatic tumors. Based on the results we performed a comparative study between OB and NOB patients to (1) assess the ELF transcriptomic profiles of each, (2) analyze the transcriptomic profiles of acinar tissue neighboring ILF (Ac/ILF+) compared to that without ILF infiltration (Ac/ILF-), and (3) characterize precancerous lesions in OB patients by morphological and immunohistochemical analyses.

## Results

### MALDI LIPIDOMIC analysis

#### Patients

Thirty patients (21 (NOB) and 9 (OB)) were included in group 1 to assess the lipidomic profiles of pancreatic ILF and ELF by MALDI-MSI and search for specific lipid peaks associated with BMI and PanIN in malignant pancreatic tumors. The clinical features are summarized in supplemental Table 1.

**Table 1:** Lipid identification with LIPID MAPS® Structure Database (Group 1).

m/z (TOF)	m/z (FTICR)	*Matched m/z : LIPID MAPS® (LMSD)*	Brut Formula	Lipid category	Lipid class
518.292*	518.32143	518.3217	C_24_H_50_NO_7_PNa	Glycerophospholipids [GP]	Glycerophosphocholines [GP01] Glycerophosphoethanolamines [GP02]
534.271*	534.29581	534.2956	C_24_H_50_NO_7_PK	Glycerophospholipids [GP]	Glycerophosphocholines [GP01] Glycerophosphoethanolamines [GP02]
558.311*#	558.29599	558.2956	C_26_H_50_NO_7_PK	Glycerophospholipids [GP]	Glycerophosphocholines [GP01]
560.315*	560.31183	560.3113	C_26_H_52_NO_7_PK	Glycerophospholipids [GP]	Glycerophosphocholines [GP01]
725.541^#^	725.55617	725.5568	C_39_H_79_N_2_O_6_PNa	Sphingolipids [SP]	Phosphosphingolipids [SP03]
726.543^#^	726.56005	726.5620	C_39_H_78_NO_8_PLi	Not identified	
728.546	728.51977	728.5201	C_38_H_76_NO_8_PNa	Glycerophospholipids [GP]	Glycerophosphocholines [GP01] Glycerophosphoethanolamines [GP02]
735.558^#^	735.57208	735.5647	C_39_H_76_NO_8_P	Not identified	
756.545^#^	756.55069	756.5514	C_40_H_80_NO_8_PNa	Glycerophospholipids [GP]	Glycerophosphocholines [GP01] Glycerophosphoethanolamines [GP02]
757.546^#^	757.55441	757.5508	C_41_H_83_O_7_PK	Glycerophospholipids [GP]	Glycerophosphates [GP10]
768.565^#^	768.55144	768.5514	C_41_H_80_NO_8_PNa	Glycerophospholipids [GP]	Glycerophosphocholines [GP01] Glycerophosphoethanolamines [GP02]
782.54^#^	782.54313	782.5460	C_42_H_82_NO_7_PK	Glycerophospholipids [GP]	Glycerophosphocholines [GP01] Glycerophosphoethanolamines [GP02]
783.542^#^	783.5531	783.5534	C_44_H_79_O_9_P	Glycerophospholipids [GP]	Glycerophosphoglycerols [GP04]
792.557^#^	792.56663	792.5668	C_44_H_84_NO_6_PK	Not identified	
830.572^#^	830.56716	830.5670	C_46_H_82_NO_8_PNa	Glycerophospholipids [GP]	Glycerophosphocholines [GP01] Glycerophosphoethanolamines [GP02] Glycerophosphoserines [GP03]
831.574^#^	831.56976	831.5706	C_40_H_80_NO_13_P	Not identified	
832.576^#^	832.58215	832.5827	C_46_H_84_NO_8_PNa	Glycerophospholipids [GP]	Glycerophosphocholines [GP01] Glycerophosphoethanolamines [GP02]
833.577^#^	833.5852	833.5878	C_46_H_83_O_10_PLi	Not identified	
834.579^#^	834.59806	834.5983	C_46_H_86_NO_8_PNa	Glycerophospholipids [GP]	Glycerophosphocholines [GP01] Glycerophosphoethanolamines [GP02]
835.628^#^	835.66682	835.6663	C_47_H_93_N_2_O_6_PNa	Sphingolipids [SP]	Phosphosphingolipids [SP03]
836.63 ^#^	836.66838	836.6610	C_49_H_89_NO_9_	Sphingolipids [SP]	Phosphosphingolipids [SP03]
Intra lobular fat (ILF)					
798.519 *	798.5214	798.5198	C_45_H_78_NO_6_PK	Not identified	
799.569*	799.56221	799.5614	C_43_H_85_O_8_PK	Glycerophospholipids [GP]	Glycerophosphates [GP10]

*Lipid-peaks specific to obesity (BMI ≥ 30).

^#^Lipid-peaks specific to the presence of PanIN lesions (PanIN +).

### Distribution of differential lipid-peaks by MALDI-TOF analysis

**Figure 1:**
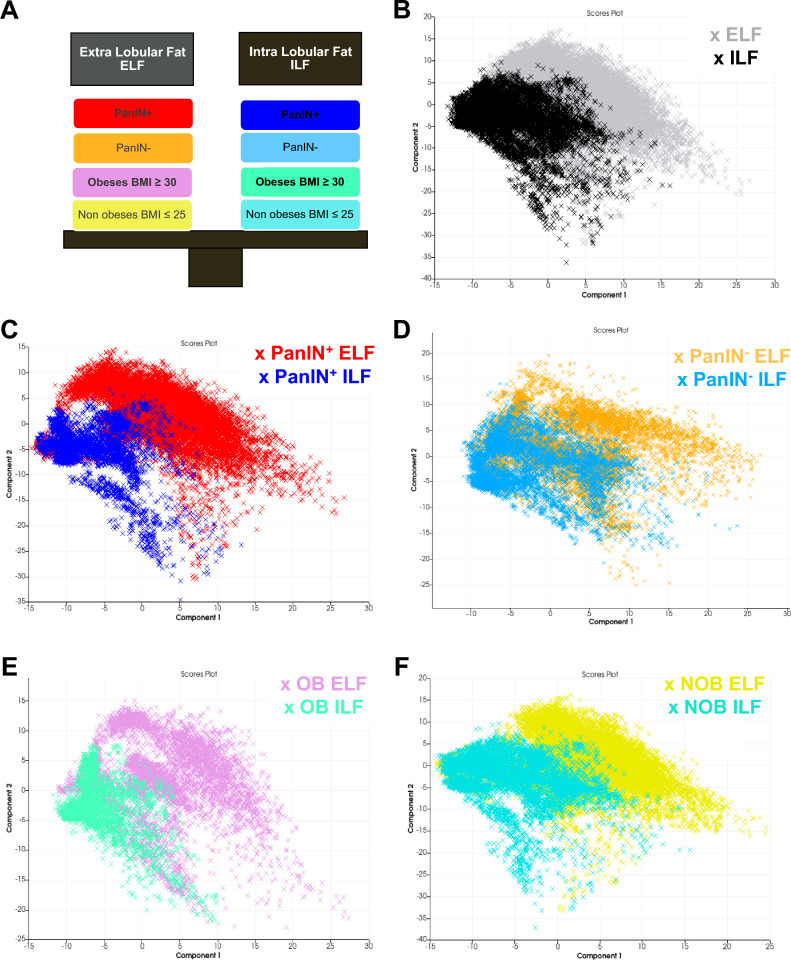
Principal component analysis (PCA) score plots of lipid peaks in OB and NOB patients. (**A**) Explanatory diagram of the different comparisons (*the colors correspond to the representation of sample groups in each PCA*). (**B**) PCA score plots of ILF (black dots) and ELF (gray dots) based on OB and NOB patient data indicating a segmentation between ILF and ELF in all patients. (**C**) PCA score plots of ILF (blue dots) and ELF (red dots) based on PanIN lesion (PanIN^+^) patient data allowing a segmentation between ILF and ELF. (**D**) PCA score plots of ILF (light blue dots) and ELF (orange dots) based on the data from patients without PanIN lesions (PanIN^-^) resulting in non-segmentation between ELF and ELF. (**E** and **F**) PCA score plots of ILF and ELF based on the BMI (OB in E and NOB in F) with a segmentation between ELF and ILF in OB patients. *PCA score plots were generated using Scils Lab Pro® software. ILF, pancreatic intralobular fat; ELF, pancreatic extralobular fat; BMI, body mass index; OB, obese patient; NOB, non-obese patient; PanIN, pancreatic intraepithelial neoplasia.*

Twenty ELF regions of interest (ROIs) and 21 ILF ROIs were selected and principal component analysis (PCA) was performed according to the BMI and the presence of PanINs (Fig. 1A). The samples were differentially distributed according to the type of fatty infiltration (ILF or ELF) (Fig. 1B) and to the presence of PanINs(PanIN^+^) (Fig. 1C). This differential distribution of ILF and ELF samples was also observed in OB patients (Fig. 1E). The difference between ILF and ELF was not evident in patients without PanIN lesions (PanIN^-^ group) (Fig. 1 D) or when the BMI was normal (BMI ≤ 25) (Fig. 1F). This suggests that the lipid composition between ILF and ELF is different in OB patients with malignant pancreatic tumors and in the presence of PanINs.

The PCA did not show any clear clusters of the same fat types (ELF or ILF) according to PanIN or BMI (supplemental Fig. 2).

**Figure 2:**
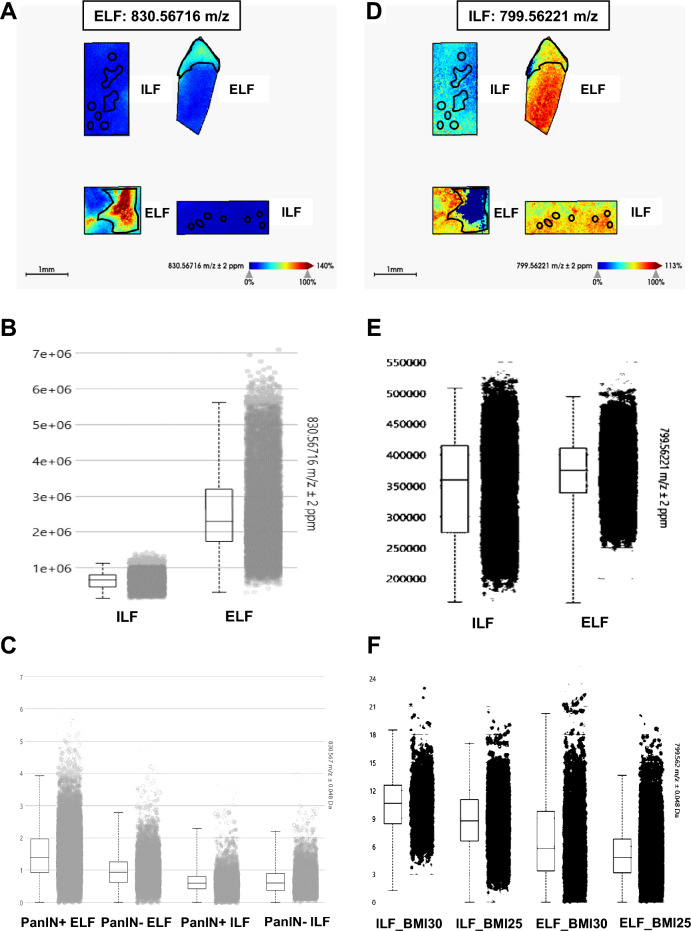
Example of the specific peaks of interest in ILF or ELF analyzed by MALDI FTICR. FTICR image of the m/z 830.56716 ELF and PanINs—specific lipid peak (**A**) and box plot comparing peaks between ILF and ELF in FTICR analysis (**B**) or in MALDI TOF analysis according to the presence or absence of PanINs (**C**). FTICR image of the m/z 799.56221 OB patient ILF-specific lipid peaks (**D**) and box plot comparing ILF and ELF in FTICR analysis (**E**) or in MALDI TOF analysis according to BMI (**F**). *ILF, pancreatic intralobular fat; ELF, pancreatic extralobular fat; PanIN, pancreatic intraepithelial neoplasia; BMI, body mass index; OB, obese patients; NOB, non-obese patients.*

### ELF and ILF present specific lipid-peaks by MALDI TOF analysis

After segmentation using SCiLS Lab Pro® software, 184 peaks were identified in this dataset. Thirty-seven of these lipid peaks were specific to ELF and 18 to ILF (AUC > 0.75). Multivariate analysis of the peaks of interest in a model including peak intensity, BMI and the presence of PanIN, was performed to identify peaks that were independently specific to fat types (ILF or ELF). Multivariate analysis identified 21 peaks specific to ELF and 2 to ILF (supplemental Table 2). The 2 ILF-specific peaks were related to BMI and were confirmed on univariate analysis (*p* = 0.04). Four ELF-specific peaks were correlated to an increased BMI (considered as a continuous value) and 17 were associated with the presence of PanINs (PanIN^+^ group), confirmed on univariate analysis (*p* < 0.05) (supplemental Table 2). The significant lipid peaks are summarized in supplemental Table 2. These results confirm that patients with malignant tumors have different ELF and ILF lipid compositions and that obesity (BMI ≥ 30) influences ILF and ELF lipid composition. Moreover, the presence of PanINs influences ELF-specific lipids, but not ILF lipid composition.

**Table 2:** Pathological characteristics of Group 2 (IHC analysis).

Pathological parameters	OB, n = 8	NOB, n = 6	*p*
Number of acinar nodules (AN)*	4 [0–16]	5 [0–11]	NS
Number of acinar-to-ductal metaplasias (ADM)*	0.5 [0–6]	0 [0–0]	0.0849
Total number of pancreatic intraepithelial neoplasias (PanIN)*	7 [0–19]	0 [0–9]	0.05
Extralobular fatty infiltration (ELF) scores*	1 [1, 2]	0 [0–0]	0.0029
Intralobular fatty infiltration (ILF) scores*	2 [1, 2]	0 [0–1]	0.0103
Fibrosis Score*	1 [0–2]	0 [0–1]	0.0924

*Quantitative data are expressed as medians and ranges.

### Lipid-peak validation on MALDI FTICR analysis and their identification using the LIPID MAPS ® database

The *m/z* confirmation was performed in situ by MALDI FTICR mass spectrometry imaging (FTICR SolariX 7T) with higher spectral resolution than the MALDI TOF analysis. Analysis with this high-resolution technique (105 to 106 FWHM) and high precision mass measurement in the sub-ppm range^17^ was more accurate.

MALDI FTICR analysis was performed to obtain more accurate lipid peak mass and the LIPID MAPS® Structure Database (LMSD, https://www.lipidmaps.org/data/structure/) was used to identify category and class.

Sixteen ELF-specific lipid peaks were identified including 5 peaks that were not identified in the LIPID MAPS database (Table 1). The ELF-specific lipid peaks were essentially glycerophospholipids (Glycerophosphocholines, Glycerophosphoethanolamines, Glycerophosphoserines or Glycerophosphoglycerols) and sphingolipids (Phosphosphingolipids). The lipid identification (*m/z*) by MALDI FTICR and LIPID MAPS database are summarized in Table 1. One example of an ELF-specific glycerophospholipid (*m/z* 830.56716) associated with the presence of PanINs is showed in Fig. 2A–C. Two ILF-specific lipid peaks were *m/z* 798.5214 (C_45_H_78_NO_6_PK) as well as *m/z* 799.56221 (C_43_H_85_O_8_PK) which was identified as a Glycerophosphate [GP10] lipid class (Table 1, Fig. 2D–2F). This lipid peak is also over-expressed in the parenchyma as showed in the FTICR figure (Fig. 2C).

Glycerophosphocholines and Glycerophosphoethanolamines in ELF, and Glycerophosphates in ILF, were specific to OB patients with malignant pancreatic tumors. Glycerophosphocholines, Glycerophosphoethanolamines, Glycerophosphoserines or Glycerophosphoglycerols) and sphingolipids (Phosphosphingolipids) specific to the ELF were related to the presence of PanIN lesions (PanIN+) in OB patients with malignant pancreatic tumors.

Based on the observation that ELF and ILF have specific lipid-peaks in patients with malignant pancreatic tumors and that ILF lipid-peaks were related to obesity, we hypothesized that the ELF and the ILF in OB patients could play a role in the initiation of precancerous pancreatic lesions (PanINs). We therefore performed transcriptomic analysis of ELF and the pancreatic parenchyma (mostly acinar cells) infiltrated or not by ILF in OB and NOB patients.

### Transcriptomic profiles of pancreatic tissue–RNA-seq analysis

#### Patient characteristics

Nine OB (BMI ≥ 30 kg/m^2^) and 13 NOB patients (BMI ≤ 25 kg/m^2^) were included in Group 2 to assess the transcriptomic profiles of acinar tissue neighboring ILF (Ac/ILF+), acinar tissue without ILF (Ac/ILF-), and ELF. The general patient characteristics are presented in supplemental Table 3.

### Lipid metabolism is altered in the ELF of obese patients

**Figure 3:**
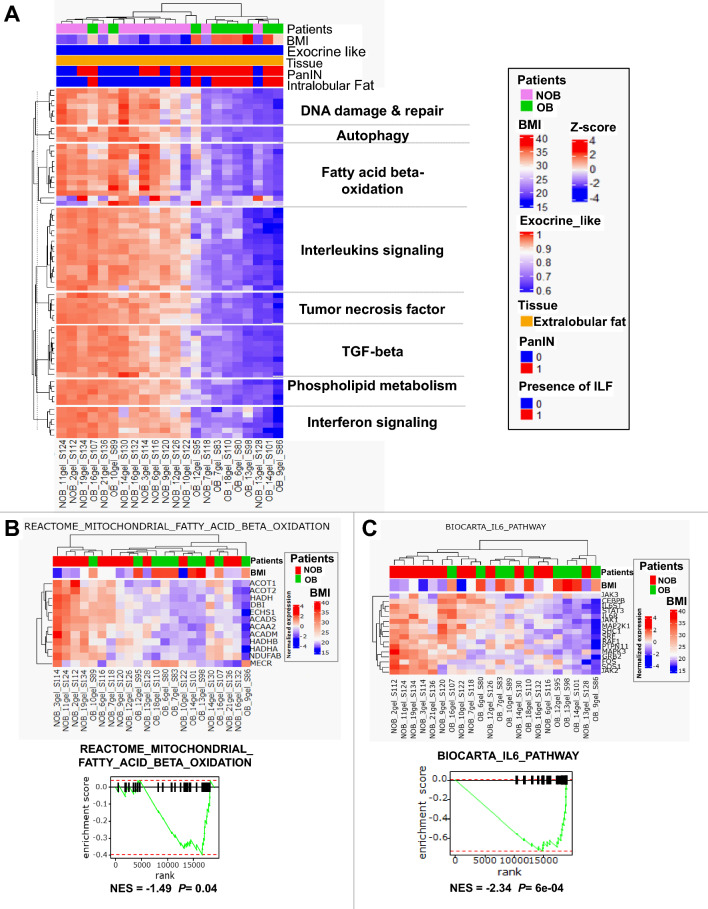
Gene set variation analysis of enriched pathways in ELF tissue. (**A**) Heat map of GSVA of pathways that were significantly enriched in the ELF tissue of NOB patients compared to OB patients. (**B** and **C**) Heat map of genes involved in beta-oxidation (**B**) and IL6 (**C**) enriched pathways and their respective leading edge. *OB, obese; NOB, non-obese; BMI, body mass index; PanIN, pancreatic intraepithelial neoplasia; ILF, pancreatic intralobular fat; ELF, pancreatic extralobular fat. NES, normalized enrichment score*.

RNA-seq analyses identified changes in ELF gene expression depending on obesity status. GSEA analysis showed significant up-regulation of NOB ELF pathways (Fig. 3A) in particular lipid metabolism pathways (especially β-oxidation of fatty acids), TGF-β receptor-signaling pathway, DNA damage and repair pathways, pro-inflammatory-signaling pathways (interleukins (IL2, IL6, IL1), interferon (α, γ) and tumor necrosis factor pathways (TNF)) and autophagy. We found that sphingolipid metabolism and fatty acid β-oxidation pathways were the most important and redundant enriched pathways in the NOB group compared to the OB group, for example mitochondrial fatty acid β-oxidation was significantly more enriched in the NOB group (NES = − 1.49, *p* = 0.04) (Fig. 3B). The inflammatory pathways that were enriched in the NOB group were pro-inflammatory signaling pathways such as interferon, TNF mediators and interleukins, in particular IL6-mediated signaling (NES = − 2.34, *p* = 6.10^–7^) (Fig. 3C). The raw gene count is provided in supplemental Table 4. These results suggest that the lipid metabolism is altered in the ELF of OB patients with benign pancreatic tumors.

### Obesity (especially ILF) enhances parenchymal (Ac/ILF+) inflammation of the pancreas in obese patients

**Figure 4:**
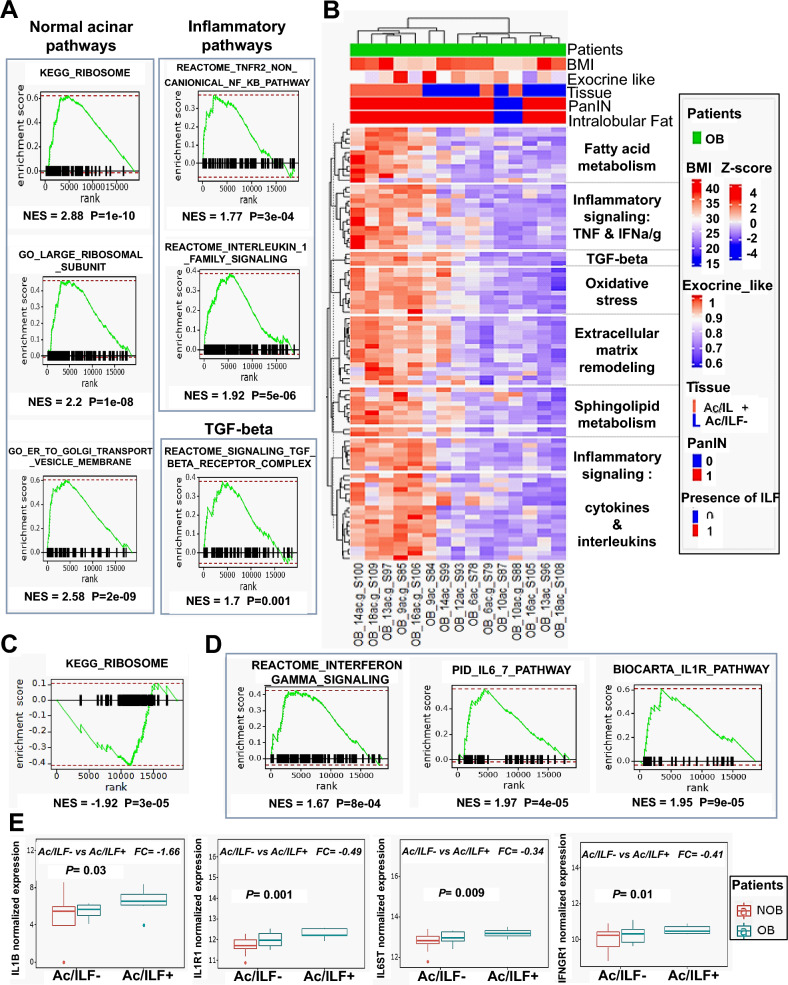
Gene set variation analysis of enriched pathways in acinar tissue. (**A**) Leading edge of enriched pathways in the Ac/ILF- of OB patients compared to NOB patients. (**B**) Heat map of the most significantly enriched pathways in Ac/ILF + compared to Ac/ILF- in OB patients. (**C**) Leading edge of enriched pathways in Ac/ILF- compared to Ac/ILF + in OB patients. (**D**) Leading edge of enriched inflammatory cytokines in Ac/ILF + compared to Ac/ILF- in OB patients. (**E**) Boxplot showing the expression of IL1B, IL1R1, IL6ST and IFNGR1 in the Ac/ILF- of OB and NOB patients and in the Ac/ILF + of OB patients. *OB, obese patients; NOB, non-obese patients; BMI, body mass index; PanIN, pancreatic intraepithelial neoplasia; NES, normalized enrichment score; ILF, pancreatic intralobular fat; Ac/ILF-, acinar tissue without ILF infiltration; Ac/ILF* + *, acinar tissue next to ILF infiltration*.

The transcriptomic profiles of acinar tissue without ILF infiltration (Ac/ILF-) were different between OB and NOB patients in relation to their metabolic and inflammatory pathways. In the OB group, Hallmark, C2 (KEGG, Reactome) and C5 (GO) databases showed TGF-β (NES = 1.7, *p* = 0.001) and inflammatory pathway enrichment in particular for interferon (IFNγ), TNFR signaling (NES = 1.77, *p* = 3.10^–4^) and some proinflammatory interleukins such as IL1 (NES = 1.92, *p* = 5.10^–6^) (Fig. 4A). Expression of ribosome and protein processing in the endoplasmic reticulum pathways (usual pathways of secretory acinar cells) was greater in the Ac/ILF- in OB patients than in that of NOB patients (Fig. 4A).

The comparisons of Ac/ILF- and Ac/ILF+ in OB patients showed upregulation of numerous pathways represented in GSVA analysis (Fig. 4B) such as: sphingolipid and fatty acid- derived biosynthetic processes, certain TGF-β-signaling pathways, oxidative stress (Reactive oxygen species, ROS) and inflammatory-signaling pathways.

The expression of ribosome pathways was greater in the Ac/ILF- in OB than in that of Ac/ILF+ (NES = − 1.92, *p* = 3.10^–5^) (Fig. 4C) as observed by comparing Ac/ILF- between NOB and OB patients. Most of the inflammatory pathways found in the Ac/ILF- of OB patients were cytokines and interleukins such as IFNG (NES = 1.67, *p* = 8.10^–4^), IL1R (NES = 1.95, *p* = 9.10^–5^), IL6 and IL7 (NES = 1.97, *p* = 4.10^–5^) (Fig. 4D).

Expression of some interleukins and their receptors/substrate such as IL1 (IL1B and IL1R1), IFNGR1 and IL6 (IL6ST) in the different acinar tissues (Ac/ILF- and Ac/ILF +) was gradually up-regulated (related to the presence of fatty infiltration) in the Ac/ILF- and the Ac/ILF + of OB patients compared to that in NOB patients (*p* < 0.05) (Fig. 4E).

There was more inflammation in the pancreatic parenchyma(acinar tissue) of OB patients with benign pancreatic tumors than in NOB patients. The increase in inflammatory pathways was greater in acinar tissue that was near to ILF infiltration (Ac/ILF +) in OB patients.

### Characterization of a new precancerous lesions of the pancreas in obese patients with benign tumors

To assess the effect of obesity on the process of the initiation of precancerous lesions of the pancreas, we performed pathological and morphological analyses of the samples of the pancreas previously analyzed by RNA-seq.

This included 14 patients (8 OB and 6 NOB patients) from group 2 (RNA-seq analysis) with complete morphological and IHC analyses.

The pathological parameters evaluated in each sample in the OB and NOB groups are summarized in Table 2.

**Figure 5:**
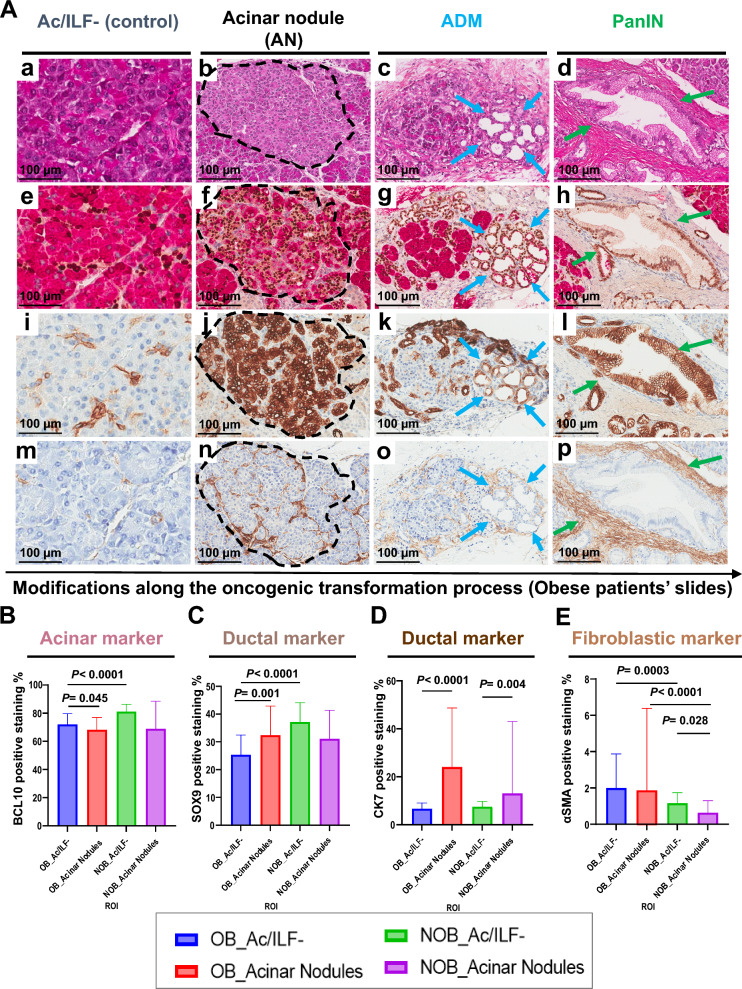
Histopathological analyses (H&E and IHC stainings) of quantified ROIs. (**A**); (**a**–**d**) H&E staining of Ac/ILF- ROIs as controls, acinar nodules (AN), ADM and PanIN lesions in OB patients. IHC staining of ROIs in and OB patient for BCL10/Sox9 (**e**–**h**), CK7 (**i**–**l**) and αSMA (**m**–**p**) of NA-control ROIs, ANs, ADM and PanIN lesions in an OB patient. (B to E) Quantification of IHC staining of BCL10 (**B**) SOX9 (**C**), CK7 (**D**) and αSMA (**E**) of Ac/ILF- and AN areas from pancreatic tissue of OB and NOB patients. *OB, obese; NOB, non-obese; ILF, pancreatic intralobular fat; Ac/ILF-, acinar tissue without ILF infiltration; AN, acinar nodule (black contouring); ADM, acinar-to-ductal metaplasia (blue arrows); PanIN, pancreatic intraepithelial neoplasia (green arrows).*

Two types of acinar areas were quantified on IHC staining (acinar, ductal and fibroblastic markers); those without ILF (Ac/ILF-) as a control, and acinar nodules (AN) (Fig. 5A). Computer-assisted quantifications (QuPath® and HALO® softwares) showed decreased expression of the acinar marker BCL10 in OB patient ANs compared to OB patient Ac/ILF- (*p* = 0.0458) (Fig. 5B). BCL10 expression was also lower in OB patient Ac/ILF- than in NOB patients (*p* < 0.0001) (Fig. 5B). In addition, the expression of the nuclear ductal marker Sox9 was greater in OB patients ANs than in OB patient Ac/ILF- (*p* = 0.0015) (Fig. 5C). Interestingly, Sox9 expression was lower in OB patient Ac/ILF- than that in NOB patients (*p* < 0.0001) (Fig. 5C).

Expression of ductal markers in acinar cells from ANs compared to that of controls (Ac/ILF-) was confirmed by the upregulation of the cytoplasmic ductal marker CK7 in OB and NOB patient ANs (*p* < 0.0001 and *p* = 0.0047, respectively) (Fig. 5D). Surprisingly, there was no difference in the expression of the fibroblastic marker (αSMA) between OB patient ANs and Ac/ILF-, and expression was reduced in NOB patient ANs compared to controls Ac/ILF- (*p* = 0.0287) (Fig. 5E). Nevertheless, αSMA expression was greater in OB patient Ac/ILF- (*p* = 0.0003) and ANs (*p* < 0.0001) than in NOB patients (Fig. 5E).

There was no difference between OB and NOB patients for the quantification of inflammatory markers throughout the parenchyma. The number of CD163 macrophages was high while the number of CD8 was low in both OB (*p* = 0.0111) and NOB (*p* = 0.0022) patients.

These results suggest that the acinar phenotype of the pancreatic parenchyma (Ac/ILF-) in OB patients is transformed in the presence of benign tumors of the pancreas. In addition, early transformation of acinar tissue into so-called “acinar nodules” (AN) is related to obesity in benign tumors of the pancreas, suggesting that AN could be the first precancerous lesion of the pancreas in OB patients.

### Precancerous epithelial lesions (PanIN, ADM, AN) and pancreatic fatty infiltration type (ILF/ELF)

The epithelial lesions that were identified were divided into early preneoplastic acinar lesions (acinar dedifferentiated, corresponding to acinar nodules (AN) and acinar-to-ductal metaplasia (ADM)), and late neoplastic lesions (PanINs). The total number of PanINs was significantly greater in OB patients than in NOB patients (*p* = 0.05) (Table 2). There was a tendency to an increased number of ADM in OB patients (*p* = 0.08) (Table 2). There was no difference in the number of ANs between OB and NOB patients. Both ILF and ELF pancreatic infiltration was significantly increased in OB patients (ELF, *p* = 0.0029 and ELF, *p* = 0.0103) (Table 2). These results show that obesity induces increased pancreatic fatty infiltration (ELF and ILF). There was no significant difference in the extent of fibrosis between OB and NOB patients (Table 2).

**Figure 6:**
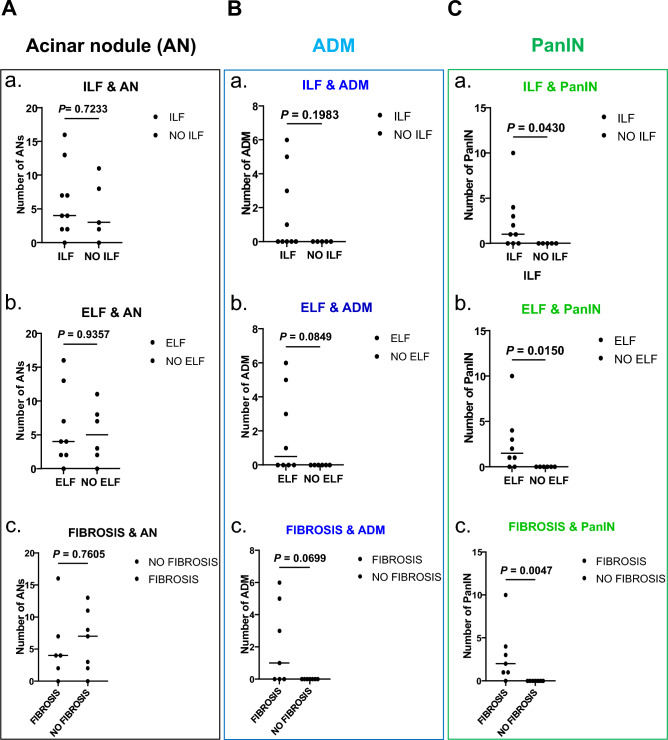
Metaplastic and neoplastic precancerous lesions with ILF/ELF and fibrosis in obese and non-obese patients. (**A**) Scatter plot analysis of the number of AN in relation to the presence/absence of ILF (**a**), ELF (**b**) and fibrosis (**c**). (**B**) Scatter plot analysis of the number of ADM lesions in relation to the presence/absence of ILF (**a**), ELF (**b**) and fibrosis (**c**). (**C**) Scatter plot analysis of the total number of PanINs in relation to the presence/absence of ILF (**a**), ELF (**b**) and fibrosis (**c**).* AN, acinar nodule; ADM, acinar-to-ductal metaplasia; PanIN, pancreatic intraepithelial neoplasia; ILF, pancreatic intralobular fat; ELF, pancreatic extralobular fat. Mann–Whitney test was performed for all the analyses.*

There was no association between ANs and ILF, ELF or fibrosis in OB or NOB patients (Fig. 6A) suggesting the very early and plastic characteristic of this lesion. The same result was observed for ADM lesions (Fig. 6B). However, there was a relationship between the total number of PanINs and pancreatic fatty infiltration. There was a significant association between the total number of PanINs and the ILF (p = 0.0430) and ELF (p = 0.015) scores (Fig. 6C,a and b). Also, the total number of PanINs was correlated to the fibrosis score (p = 0.0047) (Fig. 6C,c). These results confirm that PanINs are also related to fatty infiltration (ELF and ILF) in OB patients with benign pancreatic tumors.

## Discussion

Obesity, and especially android obesity, which is characterized by intravisceral fat infiltration, has been shown to be one of the key risk factors of PDAC. Obesity is known to be associated with pancreatic fat infiltration and two types of pancreatic fat depositions have been described, intralobular (ILF) or extralobular (ELF)^15^. This study evaluated the pathophysiological roles of ILF and ELF in the early oncogenic process in patients with obesity.

Our study found more fat infiltration, both ILF and ELF, and a higher number of PanINs in the pancreases of OB patients, confirming the impact of obesity (fatty infiltration especially) in the oncogenesis process.

We performed MALDI lipidomic analysis specifically for malignant tumors to analyze the long-term and high-risk of obesity in the development of carcinomas.

The constitutions of ILF and ELF were found to be different in all subgroup analyses, with 16 ELF-specific and 2 ILF-specific lipids despite limited ILF detection due to the lipid delocalization phenomenon and even though quantitative lipidomic analysis such as liquid chromatography MS was not performed. Nevertheless, high resolution (20µm) MALDI-MSI is interesting for in situ analysis of small areas of heterogeneous parenchyma with a few scattered adipocytes.

The lipid peaks identified in ELF were correlated with an increase in BMI (4 lipid peaks) and PanINs (17 lipid peaks) and were essentially glycerophospholipids and sphingolipids. The 2 ILF-specific lipid peaks were iGlycerophosphates [GP10], a class of lipids related to obesity (BMI ≥ 30). This supports our hypothesis that OB patient ILF could play a role in the initiation of precancerous lesions of the pancreas (PanINs). These results suggest that Glycerophosphocholines and Glycerophosphoethanolamines in ELF and Glycerophosphates in ILF are specific to OB patients presenting with malignant pancreatic tumors. Elf-specific Glycerophosphocholines, Glycerophosphoethanolamines, Glycerophosphoserines or Glycerophosphoglycerols and sphingolipids (Phosphosphingolipids) are related to the presence of PanIN lesions (PanIN+) in patients with malignant pancreatic tumors. One limit was the need of specific identification of lipid species using techniques such LCMSMS.

Lipid degradation (β-oxidation of fatty acids) and the autophagy pathways were found to be down-regulated in the ELF in OB patients on RNA sequencing, with enriched digestion of dietary lipids pathways. These results are supported by a prior lipidomic study^18^ of different tissues that showed that lipid homeostasis was altered with different lipid signatures, which made it possible to differentiate obesity and diabetes from normal tissues^18^. This is probably due to modified lipid catabolic processes causing excess lipid storage.

Our study shows that obesity and ILF infiltration influence the metabolic pathways of acinar cells and they both play a key role in the oncogenesis process. Indeed, we found that acinar tissue neighboring ILF infiltration was enriched via several pathways including lipid (sphingolipid and fatty acid- derived) biosynthetic processes, oxidative stress (Reactive oxygen species (ROS)), TGF-β and inflammatory pathways (cytokines, interleukins, IFN, TNF). This is also associated with a downregulation of ribosome pathways. This could create a stressful environment for normal acinar cells inducing the loss of secretory functions during oxidative stress and inflammation. Moreover, enrichment of inflammatory and TGF-β pathways was also found in acinar tissue without ILF infiltration in OB patients but not in NOB patients. This might be related to systemic, obesity-induced, proinflammatory effects^19–21^.

These results show that the pancreatic parenchyma (mainly acinar cells) is transformed in OB patients with benign pancreatic tumors. Acinar cells appear to be plastic during and after inflammation and to be able to transdifferentiate into duct-like cells, either directly or via an intermediate progenitor. This shows that PDAC could develop via dedifferentiation of acinar cells, before ADM and PanIN lesions^9,10,22,23^. Although this early process has not been clearly described in humans, a recent study with single-nucleus and in situ RNA-sequencing has shown that there are three different types of acinar cells that could play a role in the homeostatic and inflammatory processes of the pancreas. It has been suggested that one of these acinar populations could be linked to the development of precancerous lesions (ADM and PanINs) and PDAC^23^. This sequential process also probably plays a role in the chronic inflammation in obesity and pancreatic fat infiltration.

The functional transformation of acinar cells that was detected neighboring ILF by RNA sequencing, including the loss of secretory functions, is probably accompanied by the morphological changes resulting in so-called acinar nodules (AN), which is an early modification that occurs before the ADM-to-PanIN preneoplastic process.

The morphological approach used in this study identified a modified acinus, with an appearance on H&E stain that was different from the rest of the acinar parenchyma due to a different tinctorial quality (loss of normal basal basophilia) and characterized by a loss of acinar markers and increased ductal markers, but with no tubular structure. These AN have been reported in several studies and called acinar transformation, eosinophilic degeneration, atypical acinar cell nodules, or focal acinar cell dysplasia^12–14,24^. We characterized the ANs by immunohistochemistry in both OB and NOB patients. We found that ANs were related to obesity in patients with benign pancreatic tumors but were not associated with ILF or ELF infiltration. They are probably part of a plastic acinar transformation process. This process may be reversible but might result in ADM in a stressful and specific environment such as during inflammation or in the presence of specific lipid species.

We do not have any morphological tools to identify ANs that are at a higher risk of developing into ADM. More specific analytical tools such as single cell RNA sequencing could help. Interestingly, we found that metaplastic acinar lesions (ADM), a later event that probably corresponds to the transformation of certain acinar nodules, are more abundant in OB patients, and in areas with more fibrosis, although this was not significant (*p* = 0.086 and 0.088, respectively) probably because there were few cases analyzed. We confirm that PanINs, considered to be a later stage in the precancerous process with dysplasia, are related to fatty infiltration (either ILF and ELF) as well as pancreatic fibrosis.

One of the limits of this study, is the lack of normal pancreatic tissue as controls. There is no possibility of obtaining healthy pancreatic tissue, apart from autopsy series. The indications for pancreatic surgery are all linked to the presence of tumor or chronic pancreatitis lesions. It is also not possible to work on pancreatic biopsies which are too small. We therefore chose to work on surgical parts which are preserved and frozen for better tissue quality. Indeed, on autopsy material unfortunately a degradation of the pancreatic tissue due to the major enzymatic lysis is observed. A second consideration to take into account is the fact that different techniques and analyses were performed in our 2 groups, which represent different stages of the disease and of the pancreatic oncogenesis. This should be kept in mind in the interpretation of our results.

In conclusion, our study shows that there are two types of pancreatic fat infiltration i.e. ELF and ILF, with different lipid compositions that play different roles in the oncogenesis process, especially in OB patients. ILF plays a major role in acinar modifications and in the development of precancerous lesions in OB patients and ELF may play a role in the progression of PDAC.

## Material and methods

### Patients

We reviewed the medical files of all patients operated for pancreatic tumors in Beaujon Hospital (Tissue library of Beaujon Hospital certified NFS-96-900, Clichy, France) from January 1999 to January 2016. This tissue biobank contains both frozen samples stored at − 80 °C and FFPE tissues stored at 20 °C. An institutional review board (CEERB, comité d’éthique en recherche biomédicale du Groupe hospitalier universitaire Nord) approval for the study design and the ethical measures was obtained (IRB-00006477). An informed consent was obtained from all subjects. All methods were carried out in accordance with relevant international guidelines and regulations. The preoperative characteristics (age at surgery, BMI, metabolic syndrome, diabetes mellitus, smoking status) and the pathological features were collected.

A total of 52 patients were included in two groups according to clinicopathological data. One group underwent MALDI-TOF-MSI lipidomic analysis (Group 1, n = 30) and the other underwent RNA sequencing and histopathological analyses (Group 2, n = 22). Subgroups were defined according to BMI as obese (OB, BMI ≥ 30) or non-obese (NOB, BMI ≤ 25).

Group 1 included 30 patients (9 OB, 21 NOB) operated for malignant tumors (ampullary carcinoma, PDAC, IPMN, cholangiocarcinomas and neuroendocrine carcinomas). MALDI-TOF-MSI analysis was performed on pancreatic tissue. To be certain to remove any obstructive lesions, we selected samples at least 20 mm far from the tumor and when possible downstream the tumor. Moreover, in all cases, morphological analysis of all HE slides was performed to confirm the absence of pancreatitis lesions. We analyzed ILF and ELF compositions and searched for lipid peaks specific for pancreatic oncogenesis and malignancy.

Group 2 included 22 patients (9 OB and 13 NOB) operated for small benign, non-exocrine tumors (neuroendocrine tumors except for one solid pseudopapillary tumor). Tissues analyses were performed on pancreatic tissue without obstructive pancreatitis found downstream from the tumor.

The purpose of this group was to study early physiopathological modifications due to fat infiltration by assessing the transcriptomic profiles of acinar tissue neighboring ILF (Ac/ILF+), acinar tissue without ILF (Ac/ILF-), and of ELF from OB and NOB patients. Thus, patients with malignant tumors such as PDAC or IPMN (intraductal papillary mucinous neoplasia) were excluded.

### Pathological examination: tissue sample selection and classification

Tissue analyses were performed on samples found at a distance from the tumor on normal pancreatic tissue in all patients.

All hematoxylin–eosin-safran-stained (HES) slides were analyzed by light microscopy by a senior pathologist (AC) and two investigators (VR and SF). Investigators were blinded to the clinical data of each patient. The number of PanIN lesions was quantified. The extent and distribution of ILF and ELF was evaluated using the score developed by Klöppel et al.^25^. Briefly this score (0 to 2) was defined as the presence of fat in the interlobular space and the lobules. ELF infiltration was defined as the presence of adipocytes outside the acinar lobules, mainly located in the interlobular and peri-lobular space. Observations were scored as follows: 0: no fatty infiltration, 1: some adipocytes, and 2: numerous adipocytes separating the lobules. ILF infiltration was scored as 0: no or rare adipocytes in some lobules, 1: scattered adipocytes among most of the lobules, and 2: numerous adipocytes in most of the lobules forming clusters of more than 10 cells.

The presence of PanIN lesions was analyzed in groups 1 and 2.

In group 2, the presence of fibrosis was evaluated by a fibrosis score (0 to 3) defined as the presence of connective tissue (0: no connective tissue, 1: mild deposits of fibrosis, 2: moderate or 3: significant amounts of fibrosis) in the interlobular space and the lobules. Samples with a score ≥ 3 were considered to have significant fibrosis.

We analyzed three types of pancreatic epithelial lesions on HES and IHC sections: 1/ PanIN lesions, with either low or high grade dysplasia, 2/ Acinar-to-ductal metaplasia lesions (ADM), defined as small duct structures located in acinar tissue with an intermediate morphology between acinar cells and pancreatic ducts, expressing different intensities of ductal markers and acinar markers, 3/ acinar dedifferentiating lesions, defined by the decrease of acinar markers and/or the acquisition of ductal markers, but without ductal morphology and without any lumen formation. We called these lesions “ acinar nodules” (AN).

Specific regions of interest (ROIs) were identified by pathological analysis. In group 1 the ROIs for MALDI-TOF-MSI analysis were used to compare ILF and ELF composition between OB and NOB groups and their association to the presence of PanIN lesions (group 1, supplemental Fig. 1).

The ROIs for RNA-seq analysis corresponded to acinar tissue without ILF (Ac/ILF-), acinar tissue near ILF (Ac/ILF+), and ELF tissue (group 2, supplemental Fig. 1). In addition to late preneoplastic lesions (PanINs) and fibrosis, we analyzed ADM and AN in this group, two types of early preneoplastic acinar lesions.

### MALDI lipidomic methods–group 1

#### Tissue preparation for histology-directed MALDI TOF MSI and spectral acquisition

For each sample, a 3 µm serial cryosection, to those analyzed by MALDI-MSI, was first stained with hematoxylin–eosin (H&E). For MALDI-MSI, 10 µm thick cryosections were obtained at -17 °C using a cryostat (Leica CM1950, Leica biosystems). Each section was placed on conductive indium-tin-oxide coated slides (ITO, Bruker Daltonics, Brême, Germany). Matrix was applied by sublimation with a homemade system: 25 mg of 2,5-dihydroxybenzoic acid (DHB) matrix was dissolved with 0.5 mL of acetone, then sublimated at 120–130 °C at 6.10–2 mBar for 7 minutes^26^. The MALDI measurement was performed in positive ionization mode and with reflectron geometry using an Autoflex III MALDI-TOF mass spectrometer. The spatial resolution (Smartbeam laser) was set to 20 μm with 100 laser shots per pixel at 200 Hz. The acquisition was obtained with FlexControl 3.4 and FlexImaging 4.1 software packages (Bruker Daltonics) in the mass range from *m/z* 400 to 1200 (phospholipids mass spectra) as previously described^17^. All spectra were individually calibrated against abundant and endogenous lipids^27^ (*m/z* 796,52 [PC34:2+ K+]; *m/z* 758,56 [PC34:2+ H+]; *m/z* 824,55 [PC36:2+ K+]; *m/z* 848,55 [PC38:4+ K+]; *m/z* 575,50 [DAG-O 34 :4].

### MALDI TOF data processing

Statistical analysis was performed with SCiLS Lab® Pro 2023 (Bremen, Germany). The raw images were loaded onto the SCiLS Lab® software and baseline subtraction was performed using the convolution algorithm as well as a weak denoising. Spectra were normalized by total ion current (TIC). The peaks were then aligned using a special tool provided by Bruker®^28^. Two ROIs were determined for each case from the overlaid optical or H&E images of ILF and ELF. Segmentation was performed using the software pipeline with weak denoising for all cases to obtain a peak-list of interest.

Multivariate analysis was performed using the relative intensity of each peak from the segmentation peak-list to determine peaks that were associated with either fat type (ILF or ELF), BMI status or the presence/absence of PanINs. The significant (*p* < *0.05*) lipid peaks that were individually associated with ILF or ELF and linked to BMI or PanINs, were then kept for univariate analysis. Wilcoxon tests were used to assess the parameters directing the specific expression of each peak of interest.

Comparisons were performed in the ILF or ELF regions according to the presence or absence of PanINs and the BMI (OB or NOB).

### MALDI Fourier-transform ion cyclotron resonance (FTICR) mass spectrometry imaging

The preparation of tissue samples was the same as that described for MALDI-TOF-MSI analysis. Prepared slides were mounted on a slide adaptor (Bruker DaltoniK GmbH, Germany) and loaded into the MALDI ionization source of a 7 T FT-ICR mass spectrometer SolariX XR equipped with a Paracell (Bruker DaltoniK GmbH, Germany) and piloted by ftmsControl software, version 2.1.0 (Bruker DaltoniK GmbH, Germany) in the Protim platform in Rennes, FR (Univ Rennes, CNRS, Inserm, Biosit UAR 3480 US_S 018, Protim core facility, F-35000 Rennes, France). The instrument was set to a resolving power of 130,000 at *m/z* = 400 in the positive or negative ion mode.

The Smartbeam-II laser focus was set to “small” (20–30 μm) and a spectrum was accumulated from 100 laser shots for each pixel. The laser was run at 1000 Hz, and the ions accumulated externally (hexapole) before being transferred into the ICR cell for a single scan. Every spectrum was internally calibrated by multipoint correction in positive ([C_14_H_9_O_6_ + H]^+^ *m/z* 273.039364, [C_37_H_66_O_4_ + H]^+^ *m/z* 575.503387, [C_42_H_80_NO_8_P + H]^+^ *m/z* 758.569432, [C_42_H_82_NO_8_P + K]^+^ *m/z* 796.525313, [C_44_H_84_NO_8_P + K]^+^ *m/z* 824.556613, and [C_46_H_84_NO_8_P + K]^+^ *m/z* 848.556613). Data were acquired in the *m/z* 100–1200 mass range followed by single-zero filling and sin-apodization. Image analysis and data visualization were performed with FlexImaging 5.0 software (Bruker Daltonik GmbH, Germany). The x–y raster width, defining the lateral resolution of ion images, was set to 50 μm. On-line feature reduction was performed by ftmsControl to return only peak centroids and intensities and generate a peak list of the data in SQLite format.

### Lipid identification process : MALDI imaging analysis and metabolite annotation

After MALDI acquisition, the .mis files generated by FlexImaging from the sections were imported into the 2016b version of SCiLS Lab software (SCiLS, Bremen, Germany) using the SQLite peak list data. Analyzed sections were exported in imzML format using SCiLS Lab 2016b and submitted to the METASPACE molecular annotation engine (http://annotate.metaspace2020.eu/about,24). Metabolite annotations were assigned from four different databases (HMDB-v2.5, HMDB-v4) on the basis of accurate mass and matched isotope distributions measured with a high-resolving mass spectrometer, leading to the calculation of a metabolite signal match (MSM) score. An MSM score cut-off was applied to reach a false discovery rate of < 10%. The correlation between the theoretical *m/z* of annotations provided by Metaspace and experimental m/z of discriminant ions detected by SCiLS was performed on the basis of a Δ*m/z* < 3 ppm. When a Δ*m/z* < 3 ppm was observed between two annotations proposed by METASPACE, manual curation was performed by verifying the colocalization of the salt adducts (H^+^, Na^+^, and K^+^).

Then the lipid families were identified in a lipidomic database “Lipid Maps” LMSD (*LIPID MAPS® Structure Database, *https://www.lipidmaps.org/databases) which is a relational database encompassing structures and annotations of biologically relevant lipids.

### RNA sequencing methods–group 2

#### RNA purification from formalin-fixed paraffin-embedded (FFPE) tissue sections and sequencing

Total RNA was extracted from newly cut 9–10 μm FFPE adjacent to unstained sections using the RNeasy FFPE kit (Qiagen, France) according to the manufacturer’s protocol using 4–6 sections per case depending on the different ROIs : Ac/ILF-, Ac/ILF+ and ELF.

RNA yield and quality were determined by UV absorption on a NanoDrop One spectrophotometer (ThermoScientific®). Approximately 25µL of total RNA (1.5 ng/µL) from each sample was used for RNA Sequencing.

RNA library preparation followed manufacturer’s instructions (SMARTer Stranded Total RNA-Seq Kit v3—Pico Input Mammalian from Takara).

Final samples pooled library prep were sequenced on ILLUMINA Novaseq 6000 with S1-200 cartridge (2 × 1600Millions of 100 bases reads), corresponding to 2 × 38Millions of reads per sample after demultiplexing.

This work used equipment and services from the iGenSeq core facility (Genotyping and sequencing), at ICM (Institut du Cerveau et de la Moelle épinière, Paris).

### Bioinformatic and statistical analyzes of RNA-seq data

Raw RNA-seq reads were mapped to the human genome (Ensembl GRCH38) and Ensembl’s reference transcriptome using STAR^29^. Gene counts were obtained using FeatureCount^30^, normalized by a UpperQuartile procedure, and logged on a base 2.

RNA-seq analyses were performed using the R statistical Software (Version 4.1.2; R Core team 2021), service provided by jr-analytics.fr. Genes from sexual chromosomes and genes with null variance were removed prior to normalization. Raw Gene expression profiles were normalized using the upper-quartile approach and log2 + 1 transformed^31^.

The analysis strategy included unsupervised analysis such as Principal component analyses (PCA), as well as differential expression analysis. PCA were assessed using the 10 k genes with the highest variability across samples. Differential gene expression was estimated between groups and the statistical relevance evaluated with the Student t test. Gene set enrichment analysis (GSEA) was performed using fast GSEA implementation (10.18129/B9.bioc.fgsea; Bioconductor, open source software)^32^, pre-ranked by the Student t test value from the studied comparison. Selected pathways were assessed for single samples GSEA, or Gene Set Variation Analysis (GSVA,10.18129/B9.bioc.GSVA) and displayed as heatmaps. Independent component analyses were performed on 50% of the most variant genes from the “Acinar”- only samples or “Extralobular fat” samples separately, after gene-wise zero-centering (no unit scaling). Independent components (IC) with the strongest correlations to the presence of PanINs in histological samples, and OB vs. NOB patients were selected for further analyses. The GSEA analyses of each IC were assessed as previously described pre-ranked by each IC gene score.

A selection of significantly enriched gene sets was made between OB and NOB patients or between sample ROIs cases (Ac/ILF-, Ac/ILF + , ELF) from the GSEA databases.

### Immunohistochemical (IHC) analysis

We investigated the initial modifications that initiated precancerous lesions in the different ROIs of the exocrine pancreas.

Depending on the tissue that was available for full IHC analysis, tissue from 14 patients from group 2 (8 OB and 6 NOB) was selected for hematoxylin and eosin (H&E) slides then immunohistochemical analysis was performed on 4 µm serial sections using an automated immunohistochemical stainer according to the manufacturer’s instructions (Streptavidin-peroxidase protocol, BenchMark ULTRA, Roche®). Immunostaining was performed after dewaxing and rehydrating the slides obtained from formalin fixed and paraffine embedded samples. Antigen retrieval was performed by pretreatment at a high temperature at pH9 in TRIS Buffer. PBS was substituted for the primary antibody and used as a negative control. The slides were immunolabeled with monoclonal or polyclonal antibodies against the cytoplasmic acinar marker B-cell lymphoma 10 (BCL10, Santa Cruz Biotechnology, 1:300, sc-5273) co-immuno-stained with the nuclear ductal marker SRY-box transcription factor 9 (Sox9, Millipore, 1:600, ab5535), against the ductal marker cytokeratin 7 (CK7; Abcam, 1:400, ab125212), against alpha smooth muscle actin (αSMA; Dako, 1:600, M851) to assess activation of pancreatic stellate cells and fibroblasts, against the T-cell marker CD8 (Dako, 1:50, M7103) and the macrophage marker CD163 (Leica, 1:100, NCL-CD163). Adjacent tissues were excluded from the IHC analysis. The size and number of ROI was the same in all sample.

Fifteen areas (202,500 µm^2^/ area) of acinar tissue without ILF infiltration (Ac/ILF-) were randomly selected for each patient as “control acinar areas”, and all detectable ANs, defined by a loss of BCL10 and/or acquisition of CK7, were selected and analyzed. Immunostainings were quantified using QuPath® 0.3.2 software^33^ to assess acinar (BCL10), ductal (SOX9 and CK7) and fibroblastic (αSMA) marker expression in the control acinar areas and in AN. The total number of inflammatory cells (T lymphocytes and macrophages, CD8/CD163) was quantified throughout the parenchyma using HALO® software (v2.0.1061; 2016; Indica Labs, Inc. https://www.indicalab.com/halo/).

Statistical analyses were performed using Prism-GraphPad® software v.9.5.0.730 (GraphPad Software Inc, San Diego, CA, USA). The Mann Whitney test was performed to compare the number of precancerous lesions (AN, ADM and PanIN) per patient and the Fisher’s exact test was performed on qualitative data (ILF, ELF and fibrosis scores).

### Supplementary Information

Supplementary Figure 1.

Supplementary Figure 2.

Supplementary Table 1.

Supplementary Table 2.

Supplementary Table 3.

Supplementary Table 4.

## Data Availability

RNA sequencing analysis: Raw count data are available in supplemental data.

## Abbreviations

Ac/ILF-: Acinar tissue without ILF infiltration
Ac/ILF+: Acinar tissue next to ILF infiltration
ADM: Acinar-to-ductal metaplasia
AN: Acinar nodule
BCL10: B-cell lymphoma 10
BMI: Body mass index
CD163: Cluster of differentiation 163
CD8: Cluster of differentiation 8
CK7: Cytokeratin 7
DHB: 2,5-Dihydroxybenzoïc acid
ELF: Extralobular fat
FFPE: Formalin-fixed paraffin-embedded
FT-ICR: Fourier transform ion cyclotron resonance mass spectrometry;
GSEA: Gene set enrichment analysis
GSVA: Gene Set Variation Analysis
H&E: Hematoxylin and eosin staining
HES: Hematoxylin–eosin-safran-stained
IC: Independent component
IHC: Immunohistochemistry
ILF: Intralobular fat
IPMN: Intraductal papillary mucinous neoplasia
LMSD: LIPID MAPS® Structure Database
MALDI-MSI: Matrix-assisted laser desorption/ionization mass spectrometry imaging
PanIN: Pancreatic intra epithelial neoplasia
PBS: Phosphate buffered saline
PC: Phosphocholine
PCA: Principal component analysis
PDAC: Pancreatic adenocarcinoma
PE: Phosphoethanolamin;
PE-Cer: Ceramide phosphoethanolamin;
RNA-seq: RNA sequencing
ROI: Region of interest
SM: Sphingomyelin
Sox9: SRY-Box transcription factor 9
TG: Triglyceride
αSMA: Alpha smooth muscle actine
