# Supplementary material for: Role of the fatty pancreatic infiltration in pancreatic oncogenesis
Source: Sci Rep. 2024 Mar 19;14:6582. doi: 10.1038/s41598-024-57294-6 (PMC10951200; doi:10.1038/s41598-024-57294-6)
Supplement: Supplementary file 1 — Supplementary Figure 1. [file 41598_2024_57294_MOESM1_ESM.pdf]

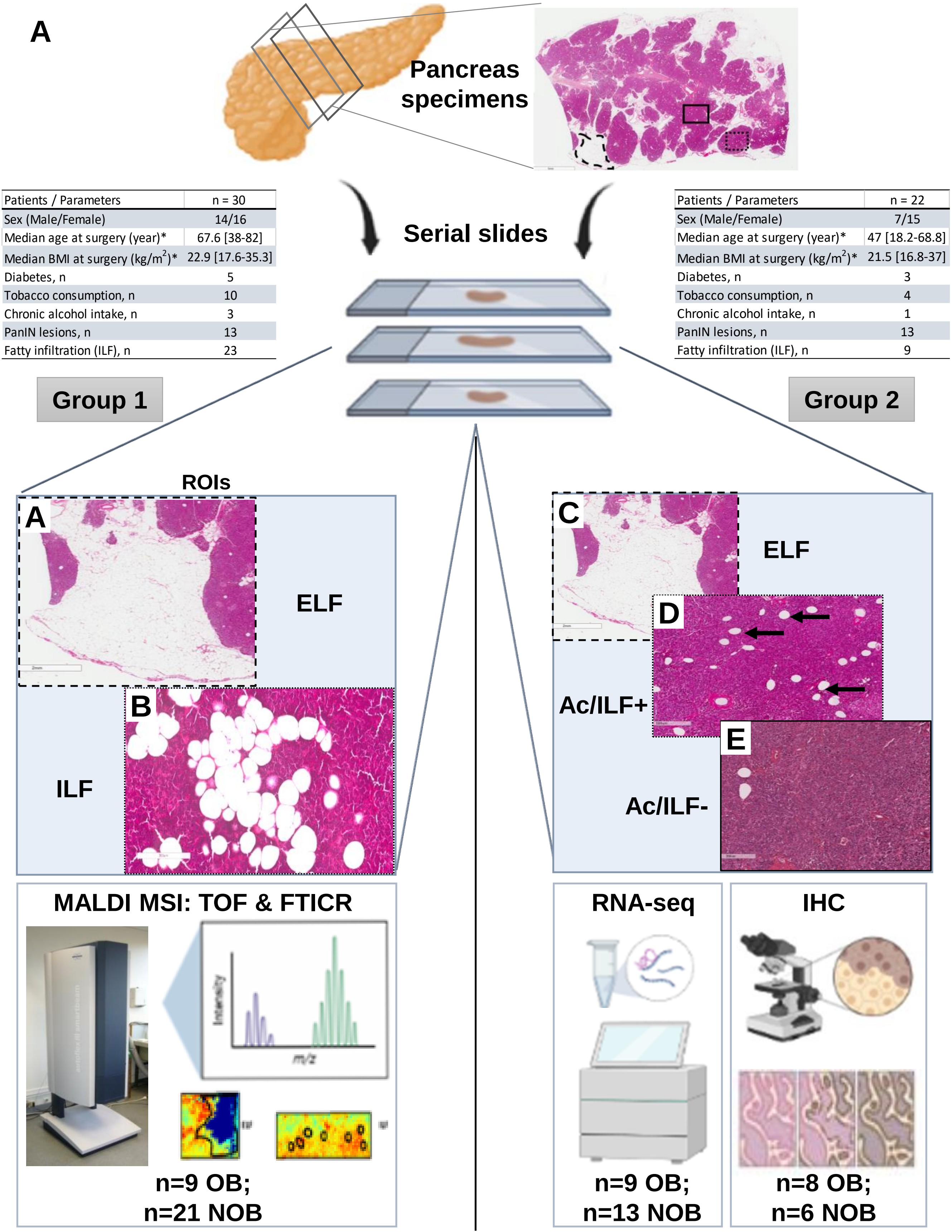

Patients / Parameters	n = 30
Sex (Male/Female)	14/16
Median age at surgery (year)*	67.6 [38-82]
Median BMI at surgery (kg/m^2)*	22.9 [17.6-35.3]
Diabetes, n	5
Tobacco consumption, n	10
Chronic alcohol intake, n	3
PanIN lesions, n	13
Fatty infiltration (ILF), n	23

Patients / Parameters	n = 22
Sex (Male/Female)	7/15
Median age at surgery (year)*	47 [18.2-68.8]
Median BMI at surgery (kg/m^2)*	21.5 [16.8-37]
Diabetes, n	3
Tobacco consumption, n	4
Chronic alcohol intake, n	1
PanIN lesions, n	13
Fatty infiltration (ILF), n	9

Supplemental figure 1. Graphic abstract of the main methods and areas selected. (A) H&E staining of pancreatic parenchyma from NOB and OB patients. Group 1 ROIs were ELF (A) and ILF (B). Group 2 ROIs were ELF tissue (C), Ac/ILF+ (D) and Ac/ILF- (E). Arrows indicate adipocytes infiltrating the parenchyma (ILF) in OB patients. *ELF, extralobular fat; ILF, intralobular fat; Ac/ILF+, acinar tissue next to ILF infiltration*; *Ac/ILF-, acinar tissue without ILF infiltration; ROIs, regions of interest; H&E, hematoxylin eosin staining; IHC, immunohistochemistry; RNA-seq, RNA sequencing; MALDI MSI, matrix-assisted laser desorption/ionization mass spectrometry imaging; TOF, time of flight; FTICR, Fourier transform ion cyclotron resonance mass spectrometry.*
