# Supplementary material for: Role of the fatty pancreatic infiltration in pancreatic oncogenesis
Source: Sci Rep. 2024 Mar 19;14:6582. doi: 10.1038/s41598-024-57294-6 (PMC10951200; doi:10.1038/s41598-024-57294-6)
Supplement: Supplementary file 2 — Supplementary Figure 2. [file 41598_2024_57294_MOESM2_ESM.pdf]

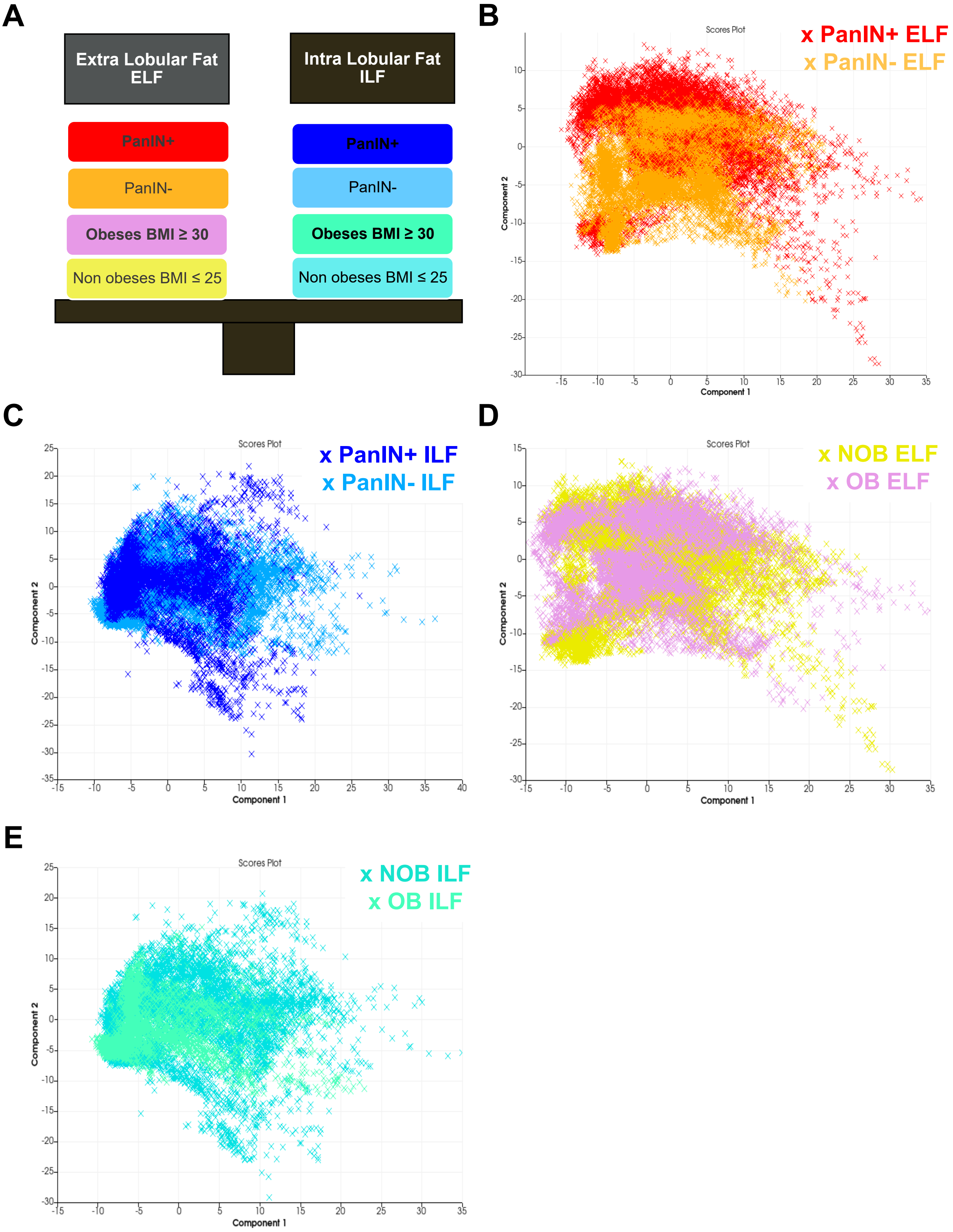
A
Extra Lobular Fat ELF
Intra Lobular Fat ILF
PanIN+
PanIN-
Obeses BMI ≥ 30
Non obeses BMI ≤ 25
PanIN+
PanIN-
Obeses BMI ≥ 30
Non obeses BMI ≤ 25
B
Scores Plot
x PanIN+ ELF
x PanIN- ELF
Component 2
Component 1
C
Scores Plot
x PanIN+ ILF
x PanIN- ILF
Component 2
Component 1
D
Scores Plot
x NOB ELF
x OB ELF
Component 2
Component 1
E
Scores Plot
x NOB ILF
x OB ILF
Component 2
Component 1

Supplemental figure 2. Principal component analysis (PCA) score plots of different patient groups in relation to the type of fat. (A) Explanatory diagram of the different comparisons (colors correspond to the examples of group samples in each PCA). PCA score plots based on the presence (PanIN$^+$) or absence of PanIN lesions (PanIN$^-$) in ELF (B) or ILF (C). PCA score plots of ELF (D) or ILF (E) based on the BMI (OB or NOB) indicating no effect of PanINs or obesity status in the same type of fat. *PCA score plots were generated using Scils Lab Pro® software. ILF, intralobular fat; ELF, extralobular fat; OB, obese patients; NOB, non-obese patients; PanIN, pancreatic intraepithelial neoplasia.*
