# Supplementary material for: Role of the fatty pancreatic infiltration in pancreatic oncogenesis
Source: Sci Rep. 2024 Mar 19;14:6582. doi: 10.1038/s41598-024-57294-6 (PMC10951200; doi:10.1038/s41598-024-57294-6)
Supplement: Supplementary file 3 — Supplementary Table 1. [file 41598_2024_57294_MOESM3_ESM.pdf]

Supplemental table 1: Group 1 Patients' characteristics (MALDI lipidomic analysis)

Patients / Parameters (n = 30)	Normal (BMI≤25), n = 21	Obese (BMI≥30), n = 9
Sex (Male/Female)	11/10	3/6
Median age at surgery (year)*	67.8 [54.4-79.4]	65.4 [38-82.4]
Median BMI at surgery (kg/m^2)*	21.5 [17-24.9]	33.5 [30-35.3]
Pancreatic ductal adenocarcinoma (PDAC), n	5	5
Ampullary carcinoma, n	7	2
Intraductal papillary mucinous neoplasm (IPMN), n	6	1
Cholangiocarcinoma, n	2	1
Neuroendocrine tumor (NET), n	1	0
Diabetes, n	2	3
Tobacco consumption, n	6	4
Chronic alcohol intake, n	3	0
Arterial hypertension, n	4	4
Hypercholesterolemia, n	2	4
Hypertriglyceridemia, n	0	0
PanIN lesions, n	9	4
Fatty infiltration (Intralobular fat), n	15	8

* Quantitative data are expressed as median and range
