# Supplementary material for: Role of the fatty pancreatic infiltration in pancreatic oncogenesis
Source: Sci Rep. 2024 Mar 19;14:6582. doi: 10.1038/s41598-024-57294-6 (PMC10951200; doi:10.1038/s41598-024-57294-6)
Supplement: Supplementary file 4 — Supplementary Table 2. [file 41598_2024_57294_MOESM4_ESM.pdf]

Supplemental table 2: Differential peaks specific to fat type identified by MALDI TOF analysis, (Group 1)

m/z (TOF)	Multivariate analysis (ANOVA)						Univariate analysis (Wilcoxon)		Fold change (mean intensity)		
	ILF ; ELF ; BMI ; PanIN						ILF or ELF only				
	Fat type		BMI		PanIN		BMI (≤25 or ≥30)	PanIN (presence or absence)	ILF/ ELF	BMI 30/25	PanIN +/-
	Weight	pValue	Weight	pValue	Weight	pValue	pValue	pValue			
Extra Lobular Fat (ELF)									ELF only		
518.292 *	-0,0795	0,1529	0,0119	**0,0196**	-0,0935	0,0955	0,2971	0,4925	0,8	2,0	0,7
534.271 *	-0,1017	0,6060	0,0485	**0,0086**	-0,3688	0,0682	0,2971	0,1016	1,3	3,7	0,4
558.311 *#	-0,0467	0,6805	0,0207	**0,0469**	-0,1757	0,1289	0,2971	**0,0169**	1,5	2,9	0,3
560.315 *	-0,0695	0,4954	0,0210	**0,0254**	-0,1561	0,1319	0,2971	0,0661	1,0	3,5	0,4
725.541 #	-0,6656	**0,0025**	-0,0143	0,4403	0,5503	**0,0112**	0,5527	**0,0229**	0,4	0,7	2,0
726.543 #	-0,2466	**0,0024**	-0,0064	0,3481	0,2103	**0,0088**	0,5520	**0,0169**	0,4	0,8	2,0
728.546	-0,1914	**0,0000**	-0,0038	0,2859	0,0994	**0,0155**	0,8624	0,0512	0,3	0,8	1,9
735.558 #	-0,0362	0,5121	-0,0002	0,9706	0,1426	**0,0134**	0,7296	**0,0355**	0,7	0,9	1,4
756.545 #	-1,0693	**0,0005**	-0,0068	0,7887	0,9258	**0,0022**	1,0241	**0,0088**	0,4	1,0	1,9
757.546 #	-0,3268	**0,0015**	-0,0049	0,5666	0,3172	**0,0020**	1,0241	**0,0088**	0,5	0,9	1,7
768.565 #	-0,1072	0,4610	-0,0026	0,8394	0,3445	**0,0225**	0,8573	**0,0088**	0,6	0,9	1,4
782.54 #	-3,4619	0,0589	0,1281	0,4243	4,1539	**0,0254**	0,8530	**0,0088**	0,7	1,1	1,4
783.542 #	-1,7996	**0,0119**	0,0267	0,6630	1,8180	**0,0114**	0,9723	**0,0088**	0,7	1,0	1,5
792.557 #	-0,1654	**0,0002**	-0,0020	0,5858	0,0865	**0,0390**	0,7352	**0,0172**	0,5	0,9	1,6
830.572 #	-0,4698	**0,0011**	-0,0171	0,1561	0,2752	**0,0453**	0,5520	**0,0352**	0,5	0,8	1,5
831.574 #	-0,1504	**0,0077**	-0,0060	0,2170	0,1135	**0,0409**	0,5520	**0,0438**	0,5	0,8	1,5
832.576 #	-0,5137	0,0536	-0,0252	0,2804	0,5926	**0,0278**	0,5520	**0,0352**	0,5	0,7	1,7

833.577 #	-0,2300	0,0742	-0,0123	0,2793	0,2684	**0,0394**	0,6093	**0,0373**	0,5	0,7	1,6
834.579 #	-0,1769	0,0716	-0,0068	0,4281	0,2445	**0,0149**	0,6686	**0,0170**	0,6	0,8	1,6
835.628 #	-0,3608	**0,0044**	-0,0131	0,2253	0,2845	**0,0225**	0,5520	**0,0355**	0,4	0,7	1,9
836.63 #	-0,1462	**0,0330**	-0,0066	0,2723	0,1493	**0,0303**	0,5520	**0,0355**	0,5	0,7	1,7
**Intra Lobular Fat (ILF)**									**ILF only**		
798.519 *	6,5497	**0,0015**	0,4604	**0,0103**	-0,3275	0,8650	**0,0430**	0,1224	1,6	1,4	0,8
799.569 *	2,2236	**0,0011**	0,1424	**0,0150**	-0,4884	0,4409	**0,0430**	0,1224	1,5	1,3	0,8

* Lipid-peaks specific to obesity (BMI ≥30)

Lipid-peaks specific to the presence of PanIN lesions (PanIN+)
