# Supplementary material for: Role of the fatty pancreatic infiltration in pancreatic oncogenesis
Source: Sci Rep. 2024 Mar 19;14:6582. doi: 10.1038/s41598-024-57294-6 (PMC10951200; doi:10.1038/s41598-024-57294-6)
Supplement: Supplementary file 5 — Supplementary Table 3. [file 41598_2024_57294_MOESM5_ESM.pdf]

Supplemental table 3: Patients' characteristics of the Group 2 (RNA-seq analysis).

**Patients / Parameters (n = 22)**	**Normal (BMI≤25), n = 13**	**Obese (BMI≥30), n = 9**
Sex (Male/Female)	3/10	4/5
Median age at surgery (year)*	43.7 [18.2-68.8]	50 [26.7-58.7]
Median BMI at surgery (kg/m^2)*	19.8 [16.8-21.5]	35 [30.1-37]
Neuroendocrine tumor (NET), n	12	9
Solid pseudo-papillary tumor of the pancreas (SPTP), n	1	0
Diabetes, n	0	3
Tobacco consumption, n	2	2
Chronic alcohol intake, n	1	0
Metabolic syndrome, n	0	6
PanIN lesions, n	5	8
Fatty infiltration (Intralobular fat), n	1	8

* Quantitative data are expressed as median and range
